# The effects and mechanisms of acupuncture for post-stroke cognitive impairment: progress and prospects

**DOI:** 10.3389/fnins.2023.1211044

**Published:** 2023-06-15

**Authors:** Ningcen Li, Hui Wang, Hang Liu, Lina Zhu, Zhongxi Lyu, Jiwen Qiu, Tianyi Zhao, Haiyan Ren, Lihong Huang, Shuangli Chen, Xiuwu Hu, Liang Zhou

**Affiliations:** ^1^Research Center of Experimental Acupuncture Science, Tianjin University of Traditional Chinese Medicine, Tianjin, China; ^2^Binhai New Area Hospital of TCM, Fourth Teaching Hospital of Tianjin University of Traditional Chinese Medicine, Tianjin, China; ^3^Xi’an Hospital of Traditional Chinese Medicine, Xi’an, Shanxi, China; ^4^Acupuncture and Moxibustion Department, Nanchang Hongdu Hospital of Traditional Chinese Medicine, Nanchang, Jiangxi, China; ^5^Acupuncture and Moxibustion Medical Clinical Research Center of Jiangxi Province, Nanchang, Jiangxi, China

**Keywords:** acupuncture, stroke, post-stroke cognitive impairment, vascular cognitive impairment, vascular dementia

## Abstract

Stroke is one of the important causes of both disability and death worldwide, which is very common in older adults. Post-stroke cognitive impairment (PSCI) is a common secondary damage of stroke, which is the main cause of long-term disability and decreased quality of life in stroke patients, which brings a heavy burden to society and families. Acupuncture, as one of the oldest and widely used worldwide techniques in Chinese medicine, is recommended by the World Health Organization (WHO) as an alternative and complementary strategy for improving stroke care. This review comprehensively summarizes literature from the last 25 years, showing that acupuncture can exert strong beneficial effect on PSCI. The mechanisms of acupuncture on PSCI involves anti-neuronal apoptosis, promoting synaptic plasticity, alleviating central and peripheral inflammatory reactions, and regulating brain energy metabolism disorders (including improving cerebral blood flow, glucose utilization and mitochondrial structure and function, etc.), etc. The effects and mechanisms of acupuncture on PSCI reviewed in this study provides scientific and reliable evidence for acupuncture application for PSCI.

## 1. Introduction

Stroke is a neurological defect caused by cerebral blood vessels, which is very common in older adults. An analysis from the Global Disease Burden Study shows that from 1990 to 2019, the absolute number of incident strokes increased by 70%. Stroke is the second largest cause of death and disability in the world after ischemic heart disease (Safiri et al., 2019). There is increasing evidence that stroke increases the risk and severity of cognitive impairment. Secondary damage to cognitive related brain regions not affected by ischemia after stroke is one of the mechanisms causing post-stroke cognitive impairment (PSCI) (Huang et al., 2022). PSCI is a common consequence of stroke, which refers to a clinical syndrome characterized by cognitive impairment that occurs after a stroke event and persists for up to 6 months. According to the severity of cognitive impairment, PSCI can be specifically divided into post-stroke cognitive impairment non-dementia (PSCIND) and post-stroke dementia (PSD) (Rost et al., 2022). PSCI is the main cause of long-term disability and decreased quality of life in stroke patients, which brings a heavy burden to society and families. PSCI occurs in approximately half of people in the first year after stroke, and incidence of cognitive impairment is nearly 50 times higher in the year after a major stroke compared with that in the general population. Even minor strokes can affect activities of daily living (ADLs), cognitive function, and quality of life (Pendlebury and Rothwell, 2019; Weaver et al., 2021). Cognitive abilities in some stroke patients can improve within the first 12 months, but gradually deteriorate thereafter. Even after 10 years, the risk of cognitive impairment persists (Guo et al., 2018). Although different subtypes of stroke, such as ischemic stroke (IS) and intracerebral hemorrhage (ICH), have different characteristics and mechanisms, they can all lead to the occurrence of PSCI (Donnellan and Werring, 2020). As the incidence and burden of stroke continues to increase, PSCI has become an increasingly serious public health challenge. Currently, there is no clear and effective treatment method for patients with PSCI, mainly focusing on delaying the further decline of cognitive impairment, improving cognitive level, psychosocial symptoms and the ability of daily living. Studies have shown that the impact of stroke related factors, such as past stroke history, stroke lesion characteristics, cerebral infarction volume, and clinical stroke severity, on PSCI risk is significant. Therefore, most international guidelines recommend that all clinical stroke patients undergo cognitive status screening before discharge (Winstein et al., 2016). However, screening for PSCI alone is far from enough. The United States Food and Drug Administration (FDA) has not yet approved therapeutic drugs for PSCI. Drugs such as donepezil, carbastin, and galantamine have certain positive effects on the relief of symptoms of PSCI, but their safety and tolerance are poor, and their clinical value is difficult to assess (Huang et al., 2022). Therefore, exploring safe and effective treatment methods to slow down the progress of PSCI is crucial.

Acupuncture is one of the oldest and widely used worldwide techniques in Chinese medicine, which is recommended by the World Health Organization (WHO) as an alternative and complementary strategy for improving stroke care (Organization, W. H., 2003). In recent years, acupuncture has shown advantages in the treatment of PSCI. Clinical trials and meta-analysis show that acupuncture is effective in improving cognitive impairment after stroke (Hung et al., 2019). In addition to significantly improving the cognitive and behavioral abilities of patients, acupuncture also has the advantage of inhibiting the risk factors of PSCI, with a potential effect of preventing the occurrence and development of PSCI. Acupuncture combined with other therapies can also have a synergistic effect on improving cognitive function. However, in recent years, few studies have systematically analyzed the potential mechanisms in this field. Therefore, in view of the need for effective management and treatment strategies for the high incidence rate of PSCI, we reviewed the basic and clinical research in the past 25 years to evaluate the role of acupuncture in preventing and treating PSCI and its possible mechanism, provide new evidence for its clinical application, and propose a promising direction for future research.

## 2. Methods

### 2.1. Search strategy

We screened the PubMed, Embase and Web of science databases for published studies, between August 1998 and March 2023 (the past 25 years). The search keywords employed were as follows: [“acupuncture” or “electroacupuncture (EA)” or “transcutaneous acupoint electrical stimulation (TAES)”] and [“post-stroke cognitive impairment (PSCI)” or “Vascular cognitive impairment (VCI)” “vascular dementia (VD)”]. A total of 647 articles in English were identified (including 423 duplicate articles).

### 2.2. Study selection

The following inclusion criteria were used for screening of the identified articles: stimulation methods included manual acupuncture (MA), EA, and TAES, and the main diseases studied included PSCI, VCI, and VD that cognitive impairment caused by stroke. We employed Excel software to manually select references that met the theme. Among them, 1 article that lacked an abstract full text, 89 articles unrelated to the theme, 62 reviews or meta-analyses and 12 articles involving the use of acupuncture in combination with other therapies, were excluded. Finally, 38 full texts of basic research articles and 22 clinical research articles, meeting the theme, were included.

**FIGURE 1:**
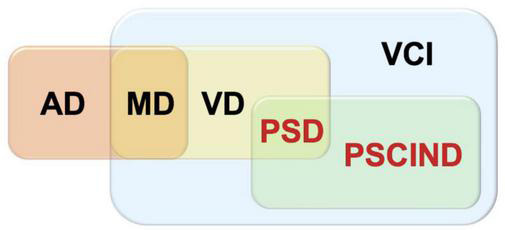
Post-stroke cognitive impairment (PSCI)-related concept diagram.

In the process of our inclusion in the articles, VCI is the most widespread concept, referring to clinical stroke or subclinical vascular injury caused by cerebrovascular diseases and their risk factors, covering all forms of cognitive impairment from vascular mild cognitive impairment (VMCI) to severe vascular cognitive impairment, as well as mixed dementia (MD) with other pathologies such as Alzheimer’s disease (AD) and VD. PSCI is included and the relationship between PSCI, VCI, and VD can be shown in the Figure 1. We only include cognitive impairment that occurs after a specific stroke event.

### 2.3. Data extraction

Due to similarities in some of the studies, we extracted key data pertaining to the theme from typical published studies, which specified the type of model, intervention (method, acupoint, and acupuncture parameters), and outcome measurement (cognitive impairment-related behavior and indicators of mechanism). Any disagreements were resolved through discussion among the authors.

## 3. Results

The pathological process of PSCI has not been fully elucidated. However, a variety of pathophysiological processes may potentially contribute to PSCI, such as such as past stroke history, stroke lesion characteristics, cerebral infarction volume, and clinical stroke severity. Numerous basic studies have shown that acupuncture improves secondary cognitive impairments, such as PSCI, mainly using the measure of improved learning memory in animal models (Yu et al., 2005). This study describes how acupuncture helps improve PSCI-related symptoms as shown in Table 1.

**TABLE 1:** Effects and mechanisms of acupuncture treating post-stroke cognitive impairment (PSCI).

References	Models	Intervention methods	Acupoints	Acupuncture parameters	Effect measurements	Biochemical measurements
Yu et al., 2005	CMi	MA	CV17, CV12, CV6, ST36, SP10	30 s, 21 days with a rest every 7 days	MWM	Escape latency↓, search strategy↑
Yang et al., 2020	BCCAO	MA	ST36, GV20	14 days (with a rest on the seventh day)	MWM	Neuronal damage↑
Wang et al., 2009	CMi	MA	CV17, CV12, CV6, ST36, SP10	Twisted at the speed of twice a second for 30 s, 210 s, 21 days (with a rest every 7 days)	MWM	Bcl-2↑, Bax↓, neuronal apoptosis↓
Liu et al., 2015	MCAO	EA	GV20, GV24	6.0 mA, 1/20 Hz, 30 min, 14 days	Neurological deficit scores, MWM	Bcl-2↑, Bax↓, neuronal apoptosis↓
Li F. et al., 2015	CMi	MA	ST36	Twisted 2 times/s for 30 s, 14 days (with a rest every 7 days)	MWM	Pyramidal neurons↑, astrocytes↓
Zhu and Zeng, 2011	BCCAO	EA	GV20, GV24, BL23	2.0 mA, 4 Hz, 20 min, 30 days	MWM	Pyramidal neurons↑, p53↓, Noxa↓
Yanzhen et al., 2008	BCCAO	EA	GV20, GV24, BL23	2.0 mA, 4 Hz, 20 min, 30 days	MWM	Neurons↑, Caspase-3↓, Noxa↓
Liu et al., 2022	BCCAO	EA	GV20, BL17, BL23	3 Hz, 10 min, 7 days	MWM	Bcl-2↑, AP-1↓, p53↓, Bax↓, JNK↓, Caspase-3↓
Feng et al., 2013	MCAO	EA	GV20, GV24	1/20 Hz, 30 min, 10 days	Neurological deficit scores, MWM	NF-κB↓, neurons↑
Ma et al., 2022	MCAO	EA	GV20, GV24	3.0 mA, 2 Hz, 30 min, 14 days	MWM	miR-81↑, IL-16↓, PSD-95↑
Xiao et al., 2018	BCCAO	MA	ST36, GV20	14 days	MWM	LTP↑, β1-AR↑, NE↑
Lin and Hsieh, 2010	MCAO	EA	GV20	2.0 mA, 100 μs, 2 Hz, 20 min	Behavior deficit score (18-point scale)	LTP↑, NMDAR1↓, TRPV1↓
Zhang et al., 2016	MCAO	EA	GV20, GV24	1/20 Hz, 30 min, 7 days	Step-down avoidance test	CaM↓, CaMKIV↑, p-CaMKIV↑, CREB↑, p-CREB↑
Dai et al., 2022	BCCAO	EA	GV20, GV24	2/20 Hz, 6 V, 1.0–3.0 mA, 30 min, 5 days/week, 4 weeks	NORT, MWM	p-NMDAR2B↑, p-GluR1↑, p-CaMKII↑
Li Q. Q. et al., 2015	CMi	MA	ST36	Turned at a rate of two spins per second for 30 s, 14 days (with a rest period every seven days)	MWM	cAMP concentration↑, PKA activity↑, pCREB↑, pERK↑
Zhu et al., 2013	BCCAO	EA	GV20, GV14, BL23	4 Hz, 2.0 mA, 20 min, 30 days	MWM	mTOR↑, eIF4E↑
Zhu et al., 2012	BCCAO	EA	GV20, GV14, BL23	4 Hz, 2.0 mA, 20 min, 30 days	MWM	p70S6↑, ribosome protein S6↑
Ye et al., 2017	BCCAO	MA	ST36, GV20	Once daily and 1 day rest after six treatments, 2 weeks	MWM	DA↑, epinephrine↑, HVA↑, D1R↑, D5R↑
Yang et al., 2014	BCCAO	MA	GV20, GV21, GV22, GV24	6 h, stimulated using the twirling method once every hour at a frequency of approximately 200 rotations per min for 3 min, 4 weeks	MWM	ACh↑, DA↑, 5-HT↑
Shao et al., 2008	MCAO	EA	GV20, BL17, BL20, BL23	150 Hz, 1.0–2.0 mA, 20 min, 15 days	MWM	AVP↑, SS↑, β-EP↑
Pan et al., 2021	BCCAO	EA	CV17, CV12, CV6, ST36, SP10	30 s, 14 days with a rest for every 7 days	MWM	IL-1β↓, IL-2 ↓, TNF-α↓, INF-γ↓, MIP-2↓, iNOS↓, COX-2↓, IL-4↑, IL-10↑, CD4^+^T↑
Qiuping et al., 2023	BCCAO	EA	CV17, CV12, CV6, ST36, SP10	30 s, 14 days with a rest for every 7 days	MWM	Th17 cells↓, Treg cell↑, CD4^+^RORγT^+^ cells↓, RORγT^+^ cells↓, CD4^+^FoxP3^+^ cells↑, FoxP3^+^cells↑
Sa et al., 2023	MCAO	EA	GV24, GV20	7 days	Neurological deficit, MWM	Hspb1↑
Yang et al., 2016	BCCAO	EA	GV20, KI3	2 Hz, 1.0 mA, 20 min, four times every other day	Y-Maze Task	Iba-1↓, TLR4↓, TNF-α↓, pERK↑, glucose metabolism↑
Wang L. et al., 2020	BCCAO	MA	ST36, GV20	2 weeks (1 day off after six treatments)	MWM	IL-6↓, TNF-α↓, TLR4↓, MyD88↓, p-NF-κB p65↓
Bu et al., 2022	4VO	EA	GV20, CV17, BL17, CV6, SP6	2/15 Hz, 1.0 mA, 30 min, 3 weeks	MWM	IL-6↓, TNF-α↓, TLR4↓, MyD88↓
Zhao et al., 2022	BCCAO	EA	ST36, GV20	–	MWM	Sirt1↑, STAT3↓, IL-17↓
Ma et al., 2020	BCCAO	MA	GV20, ST36	15 min, 2 weeks	NORT, MWM	CBF↑, myelin sheath integrity↑, IL-1β↓, IL-6↓
Zhang et al., 2014	CMi	MA	CV17, CV12, CV6, ST36, SP10	Rotated 2–3 times per second clockwise for 30 s, respectively, 21 days	MWM	CBF↑, SOD↑, CuZnSOD↑, MnSOD↑, MDA↓, superoxide anion↓, RCI↑, P/O ratio↑, mitochondrial respiratory enzymes↑
Wang et al., 2004	4VO	EA	GV20, GV14	150 Hz, 2.0 mA, 20 min, 15 days	MWM	NO↓, MDA↓, NOS↓, SOD↑, GSH-Px↑
Yang et al., 2018	BCCAO	EA	ST36, GV20	2 weeks	MWM	ROS↓, neural cell survival↑, LTP↑
Li et al., 2016	BCCAO	MA	GV20, ST36	Twisted two times per second for 30 s, 14 days (with a rest on the seventh day)	MWM	Mitochondrial respiratory complex enzymes (complex I, II, IV) ↑, cytochrome c oxidase IV↑, ROS↓
Du et al., 2018	BCCAO	MA	GV20, ST36	Twisted two times per second for 30 s, 14 days (with a rest on the seventh day)	MWM	TXNIP↓
Zhu et al., 2018	BCCAO	MA	GV20, ST36	2 weeks (1 day of rest after 6 days of treatment)	MWM	Trx-1↓, TrxR-1↓, pASK1↓, pJNK↓, p38↓
Yang et al., 2019a	CMi	MA	ST36	Twirling reinforcing manipulation, 14 weeks (with a rest every seventh day), 30 s	MWM	NF-kB↓, NF-kB p65↓, p53↓, Ca^2+,^ ROS↓
He, 2012	4VO	EA	HT9, PC9, KI1, LU11	2/100 Hz, 1.0–3.0 mA, 20 min, 14 days	Step-down apparatus	NO↓, SOD↑
Zhao et al., 2011	CMi	MA	CV17, CV12, CV6, ST36, SP10	Twirling reinforcing manipulation, 21 days (1 day rest after 6 days treatment)	MWM	Hexokinase↑, pyruvate kinase↑, glucose 6 phosphate dehydrogenase↑
Wang et al., 2021	MCAO	EA	GV20, GV24	1/20 Hz, 2.0 mA, 6 V, 30 min, 8 days	MWM	Beclin-1↑, mTOR↑, PI3K↑, p-Akt↑, p-Beclin-1↑, p-PI3K↑, p-mTOR↑, p53 mRNA↓

↑, upregulated by acupuncture; ↓, downregulated by acupuncture.

### 3.1. Anti-neuronal apoptosis

Although the pathogenesis of PSCI is complex, apoptosis has been recognized as one of the key factors for secondary injury after stroke. Cell apoptosis is triggered by internal or external stimuli, such as toxic proteins, oxidative stress, and inflammatory damage signals generated during the pathological process of PSCI may indirectly induce intracellular mitochondria and cell surface death receptor pathways, leading to neuronal apoptosis and ultimately cognitive impairment (Mattson, 2000). It is characterized by cell rounding, membrane blebbing, cytoskeletal collapse, cytoplasmic condensation, fragmentation, nuclear pyknosis and formation of membrane-enveloped apoptotic bodies, that are rapidly phagocytosed by other cells (Ghavami et al., 2014). Acupuncture can regulate the intracellular apoptotic pathway, inhibit neuronal apoptosis, and improve cognitive impairment after stroke (Yang et al., 2020). For example, MA at *Tanzhong* (CV17), *Zhongwan* (CV12), *Qihai* (CV6), *Zusanli* (ST36) and *Xuehai* (SP10) can reduce the number of apoptotic cells and the expression of proapoptotic Bax gene, increase the expression of anti-apoptotic gene B-cell lymphoma 2 (Bcl-2) in the CA1 region of the hippocampus, improve memory impairment caused by multiple cerebral infarction (Wang et al., 2009). EA at *Baihui* (GV20) and *Shenting* (GV24) can also increase the Bcl-2/Bax ratio, downregulate the number of apoptotic cells, reduce neurological deficit scores, and improve cognitive dysfunction in middle cerebral artery occlusion (MCAO) rats (Liu et al., 2015). Pyramidal neurons also play a key role in the occurrence of PSCI. MA at ST36 can significantly increase the number of pyramidal neurons in the hippocampal CA1 region, resulting in greater improvement in learning and memory abilities of microemboli saline suspension injection induced rats (Li F. et al., 2015). Further studies have shown that the process of acupuncture protecting pyramidal cells in hippocampal CA1 region may be related to inhibit the expression of p53 and its downstream Noxa (Yanzhen et al., 2008; Zhu and Zeng, 2011). During the process of cell apoptosis, on the one hand, p53 induces permeabilization of the outer mitochondrial membrane by forming a complex with a Bcl-2, resulting in cytochrome c release. On the other hand, P53 can encode a sequence specific transcription factor that controls the expression of genes that mediate cell apoptosis, such as Bax, Noxa, PUMA, Fas, and so on Chipuk et al. (2003). It is suggested that p53 and its downstream products may be another potential target for acupuncture to inhibit apoptosis and improve PSCI. EA can also upregulate the protein and mRNA expressions of Bcl-2, inhibit activator protein (AP)-1, p53, and Bax, alleviate the apoptosis of hippocampal cells in rats induced by modified bilateral common carotid artery occlusion (BCCAO), and improve their cognitive impairment, which may be related to the inhibition of JNK signaling pathway (Liu et al., 2022). The process of apoptosis is highly controlled by various intracellular pathways, including nuclear factors-κB (NF-κB) Signal conduction. NF-κB is one of the most important nuclear transcription factors. After activation, NF-κB translocates to the nucleus, where it regulates the expression of various key genes involved in apoptosis. It is reported that NF-κB is activated in a model of cognitive impairment after stroke, where NF-κB inhibitors have been shown to significantly improve cognitive function. Therefore, inhibition of NF-κB pathway may be a promising method for treating PSCI. EA at GV20 and GV24 can inhibit NF-κB activation state, thereby reducing apoptosis of brain cells and ameliorating cognitive impairment in cerebral ischemia-reperfusion injured rats (Feng et al., 2013). The above researches showed that acupuncture at CV17, CV12, CV6, ST36, SP10, GV20 and GV24 can regulate the intracellular apoptotic pathway (JNK, NF-κB), increase the Bcl-2/Bax ratio, inhibit neuronal apoptosis, and improve cognitive impairment after stroke.

### 3.2. Improving synaptic plasticity

The molecular basis of learning and memory is synaptic plasticity. Stroke can cause synaptic signal transduction and structural damage, leading to cognitive decline and memory impairment (Murphy and Corbett, 2009). Studies have shown that stroke can inhibit the expression of proteins that maintain synaptic structures (presynaptic and postsynaptic membrane scaffold proteins such as synaptophysin-1, synaptophysin and PSD-95) and proteins that regulate the integrity of synaptic functions (such as brain derived nutritional factor, microtubule associated protein-2, calcium/calmodulin dependent protein kinase 2, etc.) (Chi et al., 2023). Acupuncture can alleviate post-stroke synaptic damage, increase the number of synapses, restore synaptic morphology, structure, and transmission function, and enhance synaptic plasticity, thereby reducing cognitive impairment. For example, EA at GV20 and GV24 can increase the number of postsynaptic density protein-95 (PSD-95, an important postsynaptic protein in excitatory neurons in synapse formation) positive cells and the expression level of miR-81, inhibit the expression of IL-16 downstream of miR-81 in the frontal cortex, further improve the spatial learning and memory abilities of MCAO rats (Ma et al., 2022). Learning and memory can be achieved through the regulation of synaptic transmission intensity, and its electrophysiological basis is long-term potentiation (LTP) and long-term depression (LTD) of synaptic functional plasticity. This functional plasticity was first recorded in the glutamate neurons of CA1/3 in the rat hippocampus. Subsequently, researchers found that this phenomenon exists in multiple brain regions such as the cerebral cortex, striatum, and amygdala (Nicoll, 2017). It is found that MA at ST36 and GV20 can enhance the levels of LTP and norepinephrine (NE) in the hippocampus of rats with bilateral common carotid artery occlusion (2VO) and improve cognitive function. Meanwhile, this study found that inhibition β-adrenergic receptor (AR) instead of α-AR can block the effect of acupuncture on LTP in the hippocampus, and inhibition of β1-AR, not β2-AR, abolished the enhanced LTP, indicating the increase of NE in the hippocampus and the activation of β1-AR is a possible mechanism mediating acupuncture to improve cognitive impairment in PSCI (Xiao et al., 2018). Changes in Ca^2+^ concentration are necessary for the formation of LTP and LTD (Hell, 2016). Transient receptor potential vanilloid subtype 1 (TRPV1) is a non-selective cation channel (Ca^2+^) that is mainly expressed in sensory neurons. Its upregulation leads to increased Ca^2+^ influx, activating Ca^2+^ mediated signal transduction pathways, triggering the activation of N-methyl-d-aspartate (NMDA) receptors, promoting the movement of NMDARs, and transporting NMDARs in the cytosol to the cell membrane. The presence of extremely high concentrations of NMDARs in the brain of stroke patients can also lead to abnormal Ca^2+^ influx, resulting in an excitatory toxic cascade reaction (Huang et al., 2017). It is reported that EA at GV20 acupoint can reduce the dysfunction evoked by MCAO treatment, including behavior and LTP impairment, by downregulating NMDAR1 and TRPV1 (Lin and Hsieh, 2010). EA at GV20 and GV24 can also effectively improve cognitive ability and reduce infarction volume in MCAO rats by reducing calmodulin (CaM) activity and CaM protein expression level, upregulating the expression of calmodulin-dependent protein kinase type IV (CaMKIV), cyclic adenosine monophosphate response elements binding protein (CREB) and their phosphorylation function. The CaM-CaMKIV-CREB signaling pathway may play an important role in the regulation of PSCI by EA (Zhang et al., 2016). EA at GV20 and GV24 can downregulate the protein expression and phosphorylation levels of NMDAR2B, α-amino-3-hydroxy-5-methyl-4-isoxazolepropionic acid receptors (AMPARs) and CaMKII in the hippocampus, enhance LTP, increased the basic synaptic transmission efficiency and synaptic plasticity of the hippocampal CA3-CA1 circuit, further improving learning and memory abilities in the 2VO-induced rats (Dai et al., 2022). 3′, 5′-cyclic AMP (cAMP)/protein kinase A (PKA)/CREB has also been shown to be involved in the LTP process that mediates synaptic transmission. MA at ST36 can upregulate cAMP concentration, PKA activity, pCREB and pERK expression, and e improved hippocampal-dependent memory in rats with cerebral multi-infarction. Further blocking of PKA will reverse the improvement of recognition function caused by acupuncture treatment (Li Q. Q. et al., 2015). Some molecular pathways, such as the mammalian target of rapamycin (mTOR) signaling pathway, have been shown to promote LTP. Activating mTOR phosphorylates p70 ribosomal protein S6 (p70S6) kinase and eukaryotic translation initiation factor 4E (eIF4E) can enhance translation initiation, and plays a crucial role in the spatial learning (Bekinschtein et al., 2007; Qi et al., 2010). EA at GV20, GV14 and BL23 can also up regulate the expression of mTOR and eIF4E in the rat hippocampus, and improve the learning ability of rats (Zhu et al., 2013). EA at these acupoints can also up regulate the expression of p70S6 and ribosome protein S6, improve the learning and memory ability of 2VO-induced rats (Zhu et al., 2012). The dopaminergic system also plays an important role in regulating LTP mediated hippocampal synaptic plasticity. Studies have shown that dopamine (DA) and its related relative metabolites such as homovanillic acid (HVA) are reduced in cerebral ischemia-reperfusion rats. Acupuncture at ST36 and GV20 can promote the release of dopamine and its main metabolites (DA, epinephrine, HVA, D1R and D5R) in the hippocampus of rats, improved LTP impairment at the perforant pathway (PP)-dentate gyrus (DG) synapse, and significantly attenuated 2VO-induced learning and memory deficits (Ye et al., 2017). Acupuncture at GV20, *Qianding* (GV21), *Xinhui* (GV22) and GV24 can also upregulate the release of neurotransmitters such as acetylcholine (ACh), DA, and 5-hydroxytryptamine (5-HT) in hippocampus, improving the learning and memory functions of 2VO rats (Yang et al., 2014). In addition, some neuropeptides, such as arginine vasopressin (AVP), somatostatin (SS), and β-endorphins (β-EP), have also been shown to induce LTP enhancement and have regulatory effects on synaptic function. EA at GV20, *Geshu* (BL17), *Pishu* (BL20) and *Shenshu* (BL23) can upregulate the amount of AVP, SS, and β-EP in the plasma and brain, which is related to the improvement of learning and memory in MCAO rats (Shao et al., 2008). The above researches showed that acupuncture at ST36, GV20, GV21, GV22, GV24, BL17, BL20 and BL23 can increase the expression of proteins that maintain synaptic structures (PSD-95), enhance the levels of LTP, regulate the release of neurotransmitters/neuropeptides, enhance synaptic plasticity, and improve cognitive impairment after stroke.

### 3.3. Anti-neuroinflammation

Neuroinflammation is an important pathological mechanism of PSCI. After stroke, the levels of inflammatory cytokines in patients with cognitive abnormalities are higher than those in patients with normal cognition, such as IL-1β, IL-6, IL-8, IL-10, and IL-12, leading to nerve damage and decreased cognitive function (Narasimhalu et al., 2015). Acupuncture can help improve neuroinflammation (whether it is central or peripheral inflammatory response) after stroke and mediate the improvement of cognitive function. MA at CV17, CV12, CV6, ST36, and SP10 can increase the proportion of CD4^+^T lymphocytes in the spleen and peripheral blood, downregulate the expression of pro-inflammatory factors IL-1β, IL-2, TNF-α, INF-γ, MIP-2, COX-2, and iNOS in peripheral blood and hippocampus, upregulate the levels of IL-4 and IL-10, and improve cognitive dysfunction of BCCAO rats (Pan et al., 2021). Researchers further found that in addition to CD4^+^T lymphocytes, the Th17/Treg ratio may also affect the progression of inflammation. After cerebral ischemia, it is often observed that Treg cell activity decreases and/or Th17 cell activity increases (Kleinschnitz et al., 2013). MA at the above acupoints can also reduce the frequency and quantity of Th17 cells in these animals, increase Treg cell levels, regulate Th17/Treg balance, reduce CD4^+^RORγT^+^ and RORγT^+^ cells, increase CD4^+^FoxP3^+^ and FoxP3^+^ cell counts, thereby alleviating cognitive deficits in spatial learning and memory disorders (Qiuping et al., 2023). Toll like receptors (TLRs) are important pattern recognition receptors in the innate immune system, which initiate inflammatory cascade reactions by recognizing pathogen associated molecule pattern (PAMPs) and damage associated molecular patterns (DAMPs) (Aravalli et al., 2007). After activation, they transmit signals through the bone marrow differentiation factor 88- (MyD88-) dependent pathway to activate NF-κB transcription factor, which eventually promotes the generation of inflammatory cytokines (including IL-6 and TNF-α, etc.) (West et al., 2006). TLR4 is one of the most important members of the TLR family and plays a crucial role in inflammation after ischemic brain injury. Reducing neuroinflammation by inhibiting the TLR4 pathway may be an effective strategy for improving cognitive impairment after stroke. Some studies have found that EA at GV24 and GV20 can affect the proteomics changes of hippocampus in rats with cognitive impairment, and the up regulation of DAMPs such as heat-shock protein β1 (Hspb1) may participate in the molecular mechanism of EA to improve cognitive impairment (Sa et al., 2023). EA at *Taixi* (KI3) and GV20 can reduce the expression of neuroinflammatory proteins in the hippocampus of gerbils that had undergone BCCAO, including ionized calcium binding junction molecule 1, TLR4, TNF-α and phospho-extracellular signal-regulated kinase (pERK) to improve inflammatory response and cognitive impairment (Yang et al., 2016). MA at ST36 and GV20 can significantly downregulate the expression of TLR4, miR-93 and MyD88/NF-κB signaling pathway, inhibit the expression of inflammatory cytokines (IL-6 and TNF-α) in the hippocampus and plasma, improving cognitive function in 2VO-induced rats (Wang L. et al., 2020). In rats induced by 4VO, EA at GV20, CV17, BL17, CV6, and *Sanyinjiao* (SP6) can also inhibit the protein and mRNA expression of TLR4 and MyD88 in the hippocampus, reduce the expression of IL-6 and TNF-α in serum, promote hippocampal neuronal repair, and improve cognitive function (Bu et al., 2022). EA at GV20 and ST36 can not only inhibit TLR4 and downstream NF-κB. It can also activate the sirtuin1 (Sirt1)/signal transducer and activator of transcription 3 (STAT3) pathway, upregulate Sirt1, reduce STAT3 and inflammatory cytokines (IL-17), and improve the memory and learning abilities of VD rats (Zhao et al., 2022). TLR4 is overexpressed in microglia, but not in astrocyte and neurons. Acupuncture treatment can also down regulate the production of IL-1β and IL-6 related to microglia accumulation (Wang Z. et al., 2020). The above researches showed that acupuncture at CV17, CV12, CV6, ST36, SP10, GV24, GV20, KI3, BL17, and SP6 can regulate the state of immune cells, inhibit the activation of inflammatory pathways (TLR/MyD88/NF-κB, ERK, Sirt1/STAT3), and inhibit the release of inflammatory factors (IL-1β, IL-2, IL-6, TNF-α, INF-γ, MIP-2, COX-2, and iNOS), and improve cognitive impairment after stroke.

### 3.4. Regulating brain energy metabolism

Proper regulation of energy metabolism in the brain is crucial for maintaining brain activity under physiological and different pathophysiological conditions. Disorders in the process of energy metabolism in the brain can lead to secondary injury after stroke and deterioration of stroke outcomes, including disturbed cerebral blood circulation, mitochondrial oxidative metabolism and glucose utilization (Camandola and Mattson, 2017). They can also affect the autophagy process (Shi et al., 2021). It is found that acupuncture can regulate brain energy metabolism and improve cognitive impairment after stroke.

#### 3.4.1. Improving disturbed cerebral blood circulation

Ischemic stroke is characterized by hemodynamic changes, including sustained attenuation of cerebral blood flow (CBF) and dysfunction of CBF regulation (Ogoh, 2017). Reduced blood flow after cerebral ischemia often leads to white matter damage (a subcortical structure located deep in the non-overlapping regions of cerebral arterioles or capillaries) (Wang Z. et al., 2020). A study has used arterial spin labeling and diffusion tensor imaging (DTI) to measure CBF and white matter integrity. It has been found that BCCAO rats exhibited poor performance and changes in DTI parameters. MA at GV20 and ST36 can increase CBF, protect myelin integrity, reduce the loss of myelin basic protein, and improve cognitive impairment in BCCAO rats (Ma et al., 2020; Wang Z. et al., 2020). MA at CV17, CV12, CV6, SP10 and ST36 can also significantly increase CBF by more than 20% (Zhang et al., 2014). The above studies suggest that the recovery of cortical CBF may be one of the bases of acupuncture treatment effect.

#### 3.4.2. Improving mitochondrial dysfunction

Oxidative stress induced by ischemia may be a major risk factor in the pathogenesis of PSCI. Reactive nitrogen (RNS) and reactive oxygen species (ROS) are continuously produced in the body through mitochondrial bioenergetics and oxidative metabolism. When ROS is excessive, it can cause cumulative oxidative damage to macromolecules including DNA, proteins, and membrane lipids, disrupting antioxidant defense, directly damaging mitochondrial homeostasis and energy production, finally leading to neuronal death and cognitive dysfunction (Sies, 2017). Mitochondria are the main source of ROS and the first target of oxidative stress, and mitochondrial dysfunction can also disrupt calcium homeostasis and neural function, leading to cognitive impairment after stroke (Christophe and Nicolas, 2006). Studies have found that acupuncture can alleviate PSCI by regulating oxidative stress processes and improving mitochondrial function and structure. For example, EA at GV 20 and GV14 can significantly reduce the content of nitric oxide (NO), malondialdehyde (MDA), and NO synthase (NOS) activity, improve the activities of SOD and glutathione peroxidase (GSH-Px) regulate the production and clearance of free radicals in rats with cerebral infarction, and improve learning and memory abilities (Wang et al., 2004). EA at ST36 and GV20 can also improve cognitive functions by reducing the production of ROS and increasing the survival rate of neural cells and validated using an isobaric tag for relative and absolute quantification (iTRAQ) with high-resolution liquid chromatography-tandem mass spectrometry (LC-MS/MS) analyses (Yang et al., 2018). MA at CV17, CV12, CV6, SP10, and ST36 can also increase the activity of total SOD, CuZnSOD, and MnSOD in multi-infarct dementia (MID) rats, reduced the levels of MDA and superoxide anion, regulated the ratio of GSH and oxidized glutathione (GSSG) in mitochondria, and increased the levels of respiratory control index (RCI) and P/O ratio (Zhang et al., 2014). MA at ST36 and GV20 can significantly improve mitochondrial bioenergy parameters (mitochondrial respiratory control rate and membrane potential), increase the activity of Hippocampal mitochondrial respiratory complex enzymes (complex I, II, IV) activities and cytochrome c oxidase IV expression, which may contribute to reducing the production of ROS in the hippocampus and improving spatial learning and memory impairment (Li et al., 2016). Thioredoxin-interacting protein (TXNIP) is believed to play an important role in oxidative stress. Under oxidative stress, TXNIP oxidizes thioredoxin (Trx) by binding to the active site of Trx, enhancing oxidative stress and activating the signaling pathway mediated by apoptosis signal-regulating kinase 1 (ASK1) (Ishrat et al., 2015). MA at ST36 can improve cognitive dysfunction after stroke by reducing TXNIP related oxidative stress (Du et al., 2018). MA at ST36 and GV20 can also upregulate the expression of Trx-1 and TrxR-1, increase TrxR-1 activity, inhibit the activation of downstream ASK1-JNK/p38 pathway, inhibit oxidative stress and neuronal apoptosis damage in the hippocampus, and improve cognitive impairment caused by cerebral ischemia injury (Zhu et al., 2018). Activated immune cells produce high levels of ROS, mainly mediated by NF-κB and proinflammatory cytokines. In the presence of downstream p53, NF-κB has a proapoptotic function (Knight, 2000). MA at ST36 can inhibit the activation of NF-κB and its downstream target gene p53, inhibit the excessive production of hydroxyl free radicals and the increase of Ca^2+^ in cells, and improve the spatial learning and memory impairment induced by cerebral multi-infection (Yang et al., 2019a). Modern research has found that the subcutaneous tissue of the finger is thin and dense, with rich and dense neural sensory devices. According to the functional localization map of the primary motor and sensory areas of the cerebral cortex drawn by Penfield and Rasmussen, it can be seen that the hand occupies a large area in the somatosensory area of the cerebral cortex, and the connection between the hand and the cerebral cortex is very close. Stimulating the hand may have a greater impact on the function of the cerebral cortex than stimulating other parts. In traditional Chinese medicine, the 12 well points of the hand are located at the tip of the finger. Stimulating this point may have a greater impact on the brain than stimulating other parts (Yu et al., 2021). It is reported that the Jing-well points acupuncture can decrease the speed of the local thalamic interstitial fluid flow, down-modulate the metabolic rate of the attacked neurons under stroke in rats, which is assumed to be a beneficial protection (Fu et al., 2016). EA at related Jing-well points, such as *Shaochong* (HT9), *Zhongchong* (PC9), *Yongquan* (KI1) and *Shaoshang* (LU11) can significantly reduce the level of NO and upregulate SOD activity in the midbrain tissue and serum, enhance the ability to clear free radicals in modified 4-vessel occlusion induced rats, further improve cognitive impairment (He, 2012).

#### 3.4.3. Improving brain glucose utilization

It is often believed that glucose metabolism is almost the only source of energy in brain cells (Kuczynski et al., 2008). The activity and pathological processes in the brain of PSCI patients are related to changes in glucose metabolism. This abnormal glucose metabolism and glucose utilization that occur in the brain may occur before cognitive decline, which is an earlier pathological sign (Sato et al., 1994). Acupuncture can improve the utilization of glucose in the brain, which may have important implications for early prevention of cognitive impairment after stroke. EA treatment with KI3 or GV20 can increase glucose metabolism in the hippocampus of BCCAO mongolian gerbils and improve cognitive impairment (Yang et al., 2016). Hexokinase (HKs), the first-rate limiting enzyme involved in glucose metabolism, is considered to be one of the main regulatory steps of glycolysis in the nervous system, and its level has a good correlation with the basal metabolism rate of glucose. Glucose transported into the cell via glucose transporter (GLUT) is phosphorylated by HKs to glucose-6-phosphate (G-6-P). In addition to hexokinase, pyruvate kinase and G-6-P dehydrogenase found significant changes in specific activity in different brain regions of dementia patients (Bigl et al., 1999; Chen and Zhong, 2013). It is found that MA at CV17, CV12, CV6, SP10, and ST36 can increase the activities of hexokinase, pyruvate kinase and glucose 6 phosphate dehydrogenase, and improve the cognitive impairment of rats with MID (Zhao et al., 2011). Acupuncture may as an early preventive strategy for cognitive impairment by improving glucose utilization. In the future, the precise mechanisms of acupuncture affecting glucose metabolism in the brain can be further studied.

In addition, insufficient oxygen and glucose supply caused by cerebral ischemia can lead to an increase in AMP/ATP ratio, which can activate pathways to initiate autophagy (Shi et al., 2021). Autophagy consists of macroautophagy, microautophagy and chaperone-mediated autophagy (CMA) according to the way goods are transported to lysosomes, which helps cells eliminate unnecessary or dysfunctional components, including long-lived proteins, insoluble proteins, and even the entire organelle (Chen C. et al., 2020). Many signaling pathways are involved in the regulation of autophagy after stroke, such as phosphatidylinositol-3-kinase (PI3K)/protein kinase B (Akt) and NF-κB signal pathway (Huang et al., 2019). EA at GV24 and GV20 can activate the PI3K/Akt signaling pathway, upregulate the mRNA expression levels of related factors such as Beclin-1, mammalian rapamycin target (mTOR), and PI3K, as well as the protein expression levels of phosphorylated Akt, Beclin-1, PI3K, mTOR and autophagosomes. At the same time, it reduces the expression levels of p53 mRNA and protein, regulates the autophagy network control system, and reduces the infarct volume of rat cerebral ischemia/reperfusion injury models, finally improves learning and memory impairment in rats (Wang et al., 2021).

## 4. Discussion

Stroke is one of the diseases recommended by the WHO for acupuncture treatment (World Health Organization, 2003). A large amount of clinical evidence of traditional Chinese medicine shows that acupuncture can improve the cognitive function of stroke patients. In clinical studies, we included patients with PSCI, as well as some VD patients caused by cerebral ischemia, for analysis. Montreal Cognitive Assessment (MoCA) and Mini-Mental State Examination (MMSE) are the most commonly used evaluation scales that can quickly screen for cognitive dysfunction. MoCA and MMSE are often regarded as the primary outcome, while secondary outcomes include the neuropsychological scores (Loewenstein Occupational Therapy Cognitive Assessment, LOTCA-G), daily life function scores (Activities of Daily Living Scale, ADL; Dementia Quality of Life Questionnaire, DEMQOL; Barthel Index, BI; Modified Barthel Index, MBI; Functional activities Questionnaire, FAQ; 36-item short-form health survey, SF-36; Stroke-Specific Quality of Life Scale, SS-QOL), the Self-rating Depression Scale (SDS), Psychobehavioral scores (Hamilton Anxiety Rating Scale, HAMA; Hamilton Depression Rating Scale, HAMD; Pittsburgh Sleep Quality Index, PSQI), etc.

Acupuncture has been used for treating PSCI, adding acupuncture on the basis of routine care may have beneficial effects on improving the cognitive state and daily activities (Table 2). Acupuncture treatment schemes are often formulated according to the consensus of acupuncture experts and the preliminary research results in the earlier stage, using one or more groups of acupoints, emphasizing the role of compatibility. Acupoint prescriptions may also be customized for different patients. Neuropsychological scales such as MoCA, MMSE and LOTCA-G can be applied to the entire process of predicting the onset, early recognition, and progression of PSCI, as well as to evaluate the efficacy of clinical research. Acupuncture at GV24, GV20, GV21, GV14, *Mingmen* (GV4), BL23, *Shenmen* (HT7), *Xuanzhong* (GB39), *Sishencong* (EX-HN1), CV6, CV12, CV17, *Neiguan* (PC6), SP6, SP10, *Tianshu* (ST25), ST36, *Fenglong* (ST40), *Fengchi* (GB20), *Taichong* (LR3) can improved these scores indicating cognitive improvement (Yu et al., 2006; Huang et al., 2007b, 2021; Chou et al., 2009; Zhao et al., 2009; Shi et al., 2012, 2015; Zhang et al., 2020; Yang et al., 2022; Yusufu et al., 2022). The scalp is one of the commonly chosen areas for treatment. The scalp can be divided into seven main acupuncture areas: the parietal and frontal regions responsible for language disorders, the temporal region responsible for visual disorders, the occipital region responsible for balance disorders, the anterior parietal region responsible for swallowing disorders, and the project area responsible for memory disorders. Scalp acupuncture (SA) can also effectively improve the MoCA and MMSE scores in patients with mild vascular cognitive impairment (Chen J. et al., 2020; Jiang et al., 2020; Zhang et al., 2022). PSCI patients will have mental and behavioral abnormalities which are risk factors for mild cognitive impairment to become dementia. SA can not only improve cognitive function in PSCI patients, but also alleviates anxiety. The SDS, HAMA and HAMD were significantly improved after treatment (Huang et al., 2021; Zhang et al., 2022). Sleep disorder is also a common complication of stroke patients, which can affect the recovery of neurological function and quality of life after stroke (Swartz et al., 2016). It is positively correlated with comorbidities such as anxiety, depression, and cognitive impairment. The more severe the degree of PSCI, the higher the risk of insomnia, which can interact with each other. Therefore, ensuring good sleep is also the key to delaying the progression of PSCI. SA can improve insomnia and increase the PSQI score (Zhang et al., 2022). After stroke, patients are unable to effectively cooperate with rehabilitation training. ADL can comprehensively evaluate the physical movement, cognitive function, language, and emotional related behaviors of stroke patients. The BI and MBI focus on evaluating physical movement function. FAQ, SF-36, SS-QOL, DEMQOL, and other scales can also evaluate the quality of life of patients. Acupuncture can improve the scores of these scales (Chen, 2001; Lai and Huang, 2005; Feng, 2014; Yang et al., 2019b). In addition, acupuncture can increase intracranial blood flow rate and blood flow, improve cerebral circulation, reduce inflammatory reactions, increase brain energy metabolism, increase functional connectivity in brain regions, and thus improve cognitive level (Liu, 2004; Lun et al., 2006; Huang et al., 2007a; Shi et al., 2012; Zhi et al., 2021). The cerebral vasculature is known to be innervated by sympathetic nerves storing ATP, noradrenaline and neuropeptide Y (NPY), parasympathetic nerves with acetylcholine, vasoactive intestinal peptide (VIP), pituitary adenylate cyclase-activating peptide (PACAP), peptide histidine methionine and NO, and sensory nerves containing calcitonin gene-related peptide (CGRP), PACAP, NO, substance P (SP) and neurokinin A (NKA) (Frederiksen et al., 2019). Neurotransmitters, neurotrophic factors, and the autonomic nervous system, all of which can impact CBF, may play an important role in regulating PSCI. At present, there are few relevant studies on acupuncture to prevent and treat PSCI by regulating autonomic nerve system, but there is sufficient evidence that acupuncture can play an anti-inflammatory role by regulating vagus nerve (Li et al., 2021). In the previous summary, we also found that acupuncture can regulate neurotropism factors and improve PSCI. But research on how acupuncture regulates these processes to improve CBF and improve cognitive function can be conducted in the future. From the clinical studies we have included, acupuncture appears to be safe and effective at improving cognitive function after stroke and may have a role as a complementary therapy for PSCI. However, the evidence for its effectiveness remains limited, these results should be interpreted with caution due to the low quality of evidence. Future high-quality clinical studies are needed, including randomized doubleblind controlled trial design, large sample sizes, multiple centers, long-term efficacy evaluation, and scientific methodology, etc.

**TABLE 2:** Clinical effects of acupuncture in post-stroke cognitive impairment (PSCI).

References	Participants and methods	Intervention methods	Acupoints	Acupuncture parameters	Effect measures	Outcomes
Wang Z. et al., 2020	56 participants with PSCI	MA	Scalp	30 min, two times/day, 24 days	MoCA, MMSE, ADL, Hemoglobin	MoCA (*p* < 0.0001), ADL (*p* = 0.0019), hemoglobin↑
Lun et al., 2006	89 participants with MID	Electro-round-needling	Scalp, LI11, ST40, LR3, BL18, BL23, ST36	14–26 times/per min, 30 min, five times/week, 9 weeks	Blood rheology, NO, NOS	Blood flow↑, NO↓, NOS↓
Yusufu et al., 2022	66 participants with PSCI	MA	GV24, GV20, GV14, GV4, BL23, HT7, GB39	30 min, three times/week, 4 weeks	MMSE, MoCA, MBI, S100β, ALP	MMSE (*P*<0.05), MoCA (*P*<0.05), MBI (*P*<0.05), S100β↓, ALP↓
Shi et al., 2015	68 participants with PSCI	MA	GV20, GV24, EX-HN1, CV17, PC6, CV12, CV6, SP10, ST36, GB20, ST40, LR3, SP6, ST25	30 min, one time/2 days, 6 weeks	MMSE, ADL, DEMQOL	MMSE (*P* = 0.0141), ADL (*P* = 0.003)
Shi et al., 2014	63 participants with VD	MA	Major acupoints: GV20, EX-HN1, GV24, CV17, CV12, CV6, SP10, ST36, PC6. Adjunct acupoints: GB39, ST40, Ex-HN11, LR3, ST44, ST25, CV4	30 min, one time/2 days, 6 weeks	SDSVD	SDSVD (*P* < 0.01)
Shi et al., 2012	16 participants with VD	MA	GV20, GV24, EX-HN1, PC6, CV17, CV12, CV6, SP10, ST36, GB39, ST30, LR3, ST44, ST25, CV4	30 min, one time/2 days, 6 weeks	MMSE-R, ADL-R, DEMQOL, SDSVD, glucose metabolism	MMSE-R (*P* < 0.05), DEMQOL (*P* < 0.05), glucose metabolism↑
Zhao et al., 2009	90 participants with VD	EA	GV20, GV24, EX-HN1, GB20	300–500/min, continuous wave, 30 min, five times/week, 6 weeks	MMSE	MMSE (*P* < 0.01)
Liu, 2004	46 participants with MID	MA	GV20, GV24, GB13, HT7, LU7, KI6, GB39, SP6, ST36, GB20, GV16, GV14, LI4, LI11, SI5, LR3	Manipulate/10 min, 30 min, five times/week, 8 weeks	Blood lipid, microcirculation, capillary loops	Microcirculation (*P* < 0.01), capillary loops (*P* < 0. 05 or *P* < 0. 01), TC↓, TG↓, LDL-C↓,HDL-C↑, ApoA_1_↑, ApoB_100_↓
Yang et al., 2022	70 participants with PSCI	MA	GV20, GV16, BL11, ST37, ST39, GV14, ST36	Manipulate once every 10 min, 30 min, three times/week, 9 weeks	SDSVD, MMSE, MoCA	MMSE (*P* < 0.05), MoCA (*P* < 0.05), SDSVD (*P* < 0.05)
Jiang et al., 2020	63 participants with VD	SA	GB20, GV20, GB6, GB19, EX-HN1 GV17, GV16,	Twirle once every 10, 30 min, 60 days	RR, MMSE, ADL	RR (*P* < 0.05), MMSE (*P* < 0.05), ADL (*P* < 0.05)
Chen, 2001	32 participants with VD	MA	GV20, GV16, GV26, LI11, HT7, SP6, KI3, LR3	40 days	CCSE, FAQ	CCSE (*P* < 0.01), FAQ (*P* < 0.01)
Feng, 2014	42 participants with VD	EA	GV24, GB13, EX-B2	Continuous wave, 15 min, five times/week, 4 weeks	ADL	ADL (*P* < 0.01)
Zhi et al., 2021	56 participants with PSCI	MA	CV17, CV12, CV6, SP10, ST36, SJ5	20 min, six times/week, 12 weeks	CD3 + T, CD4 + T, IFN-γ, TNF-α	CD3^+^T↑, CD4^+^T↑, IFN-γ↑, TNF-α↓
Yang et al., 2019b	216 participants with VCID	MA	ST36, SP10, CV17, CV12, CV6, GV20, GV16, BL15, BL45, HT5, KI6, KI3, PC6, GB39, ST40, BL17	30 min, two times/week, 12 weeks	ADAS-cog, CDT, ADL	ADAS-cog (*P* = *0.002*)
Huang et al., 2007a	10 participants with VaD	MA	LI15, SP10, SJ5, LI4, ST36, SP6, LR3, GV20, GV26, HT7	stimulation/5 min, 20 min, five times/week, 4 weeks	Glucose metabolism	Glucose metabolism↑
Yu et al., 2006	60 participants with VaD	MA	CV17, CV12, CV6, ST36, SP10	Manipulate/10 min, 30 min, seven times/week, 6 weeks	MMSE, HDS-R, ADL	MMSE (*P* < *0. 05*), HDS-R (*P* < *0. 05*)
Lai and Huang, 2005	50 participants with VD	MA	LI15, LI11, SJ5, LI4, SP10, ST36, SP6, GV20, GV26, HT7	Manipulate/5 min, 20 min, five times/week, 4 weeks	HDS-R, ADL, FAQ	HDS-R, FAQ (all *P* < 0. 05 or *P* < 0.01)
Chou et al., 2009	33 participants with PSCI	EA	PC6, HT7	10–30 mA, 1 Hz, 20 min, two times/week, 8 weeks	LOTCA-G, SF-36, SS-QOL	LOTCA-G: orientation, perception, praxis, and attention (*P* < 0.05), SF-36 (RP, VT, SF, RE, MH, MCS) (*P* < 0.05), SS-QOL (language) (*P* < 0.05), LOTCA-G (*P* < 0.05)
Huang et al., 2007b	50 participants with VD	MA	GV20, GV26, HT7	Run/5 min, 20 min, five times/week, 4 weeks	MMSE, ADL, FAQ	MMSE, ADL, FAQ (all *P* < 0. 05 or *P* < 0.01)
Gao et al., 2001	63 participants with VD	MA	GV26, PC6, SP6, ST40, EX-HN1	15 min, 5 times/week, 8 weeks	HDS, P_300_, SOD, LPO	HDS (*P* < 0.01), P_3_ latency↓, P_3_ wave amplitude↑, SOD↑, LPO↓
Lai et al., 1998	46 participants with VaD	EA	GB20, PC6, EX-HN1, GB39, GV16, BL18, BL23, ST36, SP6, KI3, LR3, LR2, GB43, HR7, CV12, SP10, LI4, LI15, LI11, SJ5, GB30, GB34, ST4, ST6, GB14, ST1	Continuous wave,120-250 frequency/min, 30 min, 5 times/week, 7 weeks	HDS, FAQ, SOD, LPO, NO, HDS, FAQ, NDS	HDS (*P* < 0.01), FAQ (*P* < 0.01), SOD↑, LPO↓, NO↓
Zhang et al., 2022	660 participants with PSCI	IDSA	MS6, GV21, GB6, MS7, GV20, GB7	0.25–0.35 deep, 30 min, six times/week, 8 weeks	MMSE, MoCA, HAMD, HAMA, PSQI, MBI	MMSE (*P* < 0.01), MoCA (*P* < 0.01), HAMD (*P* < 0.01), HAMA (*P* < 0.01), PSQI (*P* < 0.01), MBI (*P* < 0.01)

↑, upregulated by acupuncture; ↓, downregulated by acupuncture.

PSCI, post-stroke cognitive impairment; PSCIND, post-stroke cognitive impairment non-dementia; PSD, post-stroke dementia; ADLs, activities of daily living; IS, ischemic stroke; ICH, intracerebral hemorrhage; FDA, Food and Drug Administration; WHO, World Health Organization; TEAS, transcutaneous acupoint electrical stimulation; VCI, Vascular cognitive impairment; VD, vascular dementia; MA, manual acupuncture; EA, electroacupuncture; SA, scalp acupuncture; VMCI, vascular mild cognitive impairment; MD, mixed dementia; AD, Alzheimer’s disease; Bcl-2, B-cell lymphoma 2; MCAO, middle cerebral artery occlusion; BCCAO, bilateral common carotid artery occlusion; NF-κB, nuclear factors-κB; PSD-95, postsynaptic density protein-95; LTP, long-term potentiation; LTD, long-term depression; NE, norepinephrine; AR, adrenergic receptor; TRPV1, transient receptor potential vanilloid subtype 1; NMDA, N-methyl-d-aspartate; CMi, cerebral multi-infarction; NMDAR, N-methyl-d-aspartate receptor; CaM, calmodulin; CaMKIV, calmodulin-dependent protein kinase type IV; CREB, cyclic adenosine monophosphate response elements binding protein; AMPARs, α-amino-3-hydroxy-5-methyl-4-isoxazolepropionic acid receptors; mTOR, mammalian target of rapamycin; 2VO, two-vessel occlusion; 4VO, modified 4-vessel occlusion; p70S6, p70 ribosomal protein S6; eIF4E, eukaryotic translation initiation factor 4E; DA, dopamine; HVA, homovanillic acid; PP, perforant pathway; DG, dentate gyrus; Ach, acetylcholine; 5-HT, 5-hydroxytryptamine; TLRs, Toll like receptors; PAMPs, pathogen associated molecule pattern; DAMPs, damage associated molecular patterns; MyD88, bone marrow differentiation factor 88; Hspb1, heat-shock protein β1; pERK, phospho-extracellular signal-regulated kinase; Sirt1, sirtuin1; STAT3, signal transducer and activator of transcription 3; CBF, cerebral blood flow; DTI, diffusion tensor imaging; RNS, reactive nitrogen; ROS, reactive oxygen species; NO, nitric oxide; MDA, malondialdehyde; NOS, NO synthase; GSH-Px, glutathione peroxidase; MID, multi-infarct dementia; GSSG, oxidized glutathione; RCI, respiratory control index; TXNIP, thioredoxin-interacting protein; Trx, thioredoxin; MoCA, Montreal Cognitive Assessment; MMSE, Mini-Mental State Examination; MBI, Modified Barthel Index; SDS, Self-rating Depression Scale; ADL, Activities of Daily Living Scale; DEMQOL, dementia quality of life questionnaire; SDSVD, scale of differentiation of syndromes of vascular dementia; FAQ, Functional activities Questionnaire; HDS-R, Hasegawa’s dementia scale; LOTCA-G, Loewenstein Occupational Therapy Cognitive Assessment; SF-36, 36-item short-form health survey; SS-QOL, Stroke-Specific Quality of Life Scale; SOD, suoeroxide dismutase; LOP, lipid peroxide; NDS, neurologic deficit soring; HAMD, Hamilton Depression Rating Scale; HAMA, Hamilton Anxiety Rating Scale; PSQI, Pittsburgh Sleep Quality Index. CV4, *Guanyuan*; CV6, *Qihai*; CV12, *Zhongwan*; CV17, *Tanzhong*;ST25, *Tianshu*; ST36, *Zusanli*; ST37, *Shangjuxu*; ST38, *Xiajuxu*; ST40, *Fenglong*;ST44, *Neiting*; SI5, *Yanggu*; SJ5, *Waiguan*; SP6, *Sanyinjiao*; SP10, *Xuehai*; GB13, *Benshen*; GB39, *Xuanzhong*; GV4, *Mingmen*; GV14, *Daizhui*; GV16, *Fengfu*; GV20, *Baihui*; GV21, *Qianding*; GV22, *Xinhui*; GV24, *Shenting*; GV26, *Renzhong*; BL11, *Dazhu*; BL17, *Geshu*; BL20, *Pishu*; BL23, *Shenshu*; BL18, *Ganshu*; KI3, *Taixi*; KI1, *Yongquan*; KI6, *Zhaohai*; HT7, *Shenmen*; HT9, *Shaochong*; PC6, *Neiguan*; PC9, *Zhongchong*; LU7, *Lieque*; LU11, *Shaoshang*; LI4, *Hegu*; LI11, *Quchi*;LI15, *Jianyu*; LR2, *Xingjian*; LR3, *Taichong*; EX-HN1, *Sishencong*; EX-HN11,:*Shexia*; EX-B2, *Jiaji*; MS6, *anterior oblique line of vertex-temporal*; MS7, *posterior oblique line of vertex-temporal*.

In the mechanism part of acupuncture treatment for PSCI, the thalamus and hippocampus are important cognitive related brain structures. Information input from the dorsomedial thalamic nucleus can enhance cortical functional connectivity and participate in cognitive processes such as attention, executive ability and working memory. Due to the important role of the thalamus in cognition, the concept of “cognitive thalamus” has recently been proposed (Wolff and Vann, 2019). The hippocampus has always been considered a key brain area for learning and memory (Bettio et al., 2017). Therefore, secondary damage to these distant brain regions not only directly affects the recovery of sensory and motor function, but also leads to cognitive impairment. In this review, it is found that acupuncture can reach the effect of improving cognitive impairment after stroke by regulating multiple pathways in the thalamus and hippocampus (such as the ways of anti-apoptosis, anti-inflammation, promoting synaptic plasticity, and regulating brain energy metabolism, etc.). In addition to the pathological aspects of the central nervous system, acupuncture can also improve the peripheral inflammatory response after PSCI, playing a therapeutic role in PSCI. The multitarget mechanism of acupuncture indicate that acupuncture has the function of regulating the whole body. These results suggested that acupuncture has potential therapeutic and brain-protective effects and may be helpful for treating PSCI.

It is worth noting that the effects of acupuncture on PSCI are related to the selections of acupoints and manipulation of acupuncture. Scalp is an important area for acupuncture to improve the neurological function of PSCI patients. In recent years, scalp acupuncture has played an important role in increasing the blood supply of the cerebral cortex, thereby improving the metabolic rate of neurons and promoting the formation of synapses between brain cells. Scalp acupuncture therapy is widely popular in Asia due to its simple application, safety, and effectiveness (Hsing et al., 2012). GV20 and GV24 are the most commonly used acupuncture points, and GV21 and GV22 on the head are also commonly used in combination. Scalp point cluster-needling may be a potential therapeutic application for improving symptoms of PSCI patients. ST36 in the lower limbs is also the most commonly used acupoint. ST36 has a tonifying effect that can calm and regulate the mind and has a benign regulatory effect. In addition, it is reported that the Jing-well points acupuncture may assumed to be a beneficial protection, which can be received further attention in the future. The combination of acupoints often has better therapeutic effects than the individual use of some single acupoints. To observe the therapeutic effect of acupuncture and to find the most effective point combinations, some studies have compared the effects of ST36 + GV20, GV20 + GV24, and ST36 + SP10 on the improvement of cognitive function in 2VO-induced rats. It has been found that ST36 + GV20 points are the most effective combinations (Ye et al., 2017). In another study, it was also found that the combination of GV20 + ST36 has better attenuating effects on increasing CBF and improving cognitive impairment (Ma et al., 2020). Manual manipulation is another key factor that influences the therapeutic effects of acupuncture. Most research has focused on acupoint specificity, less on exploring the effect of acupuncture manipulation. Therefore, some studies have also compared the effects of different MA stimulation methods on cognitive impairment and found that the approach of needle retention with 10 min, rotation for 30 s every 5 min or daily treatment with acupuncture was more effective than non-retention, non-rotation, or alternative day treatment group (Yang et al., 2020). But these still require extensive and repetitive experiments to verify.

The intervention time of acupuncture is also important during the treatment process. Cognitive impairment is a common sequela of stroke patients, which requires a longer recovery cycle due to the compression of the central nervous system by blood clots. In traditional beliefs, cognitive impairment caused by stroke requires natural recovery. In order to accelerate the recovery process, many patients choose to undergo traditional Chinese medicine therapy after Western medicine treatment. However, this time is often a bit late, missing the best time for early intervention. During the early rehabilitation treatment of patients with cerebral infarction, acupuncture treatment can effectively promote the self-repair of the damaged cerebral cortex and better restore cognitive function.

## 5. Conclusion

**FIGURE 2:**
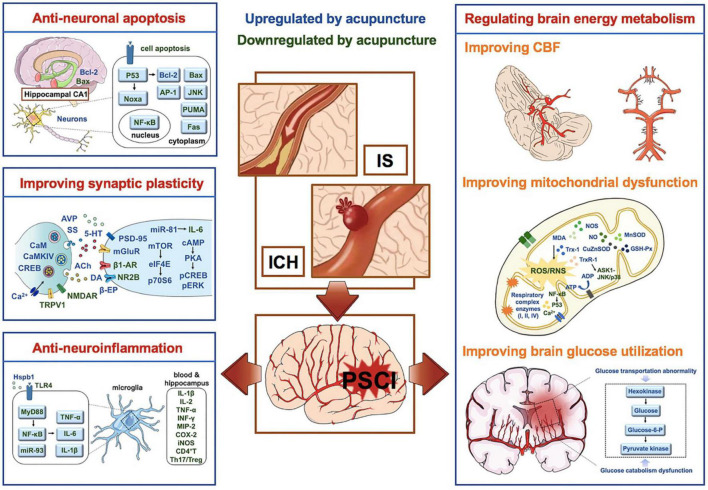
Mechanisms of acupuncture on PSCI. Factors in blue are upregulated by acupuncture, while factors in green are down-regulated by acupuncture. PSCI, post-stroke cognitive impairment; IS, ischemic stroke; ICH, intracerebral hemorrhage; Bcl-2, B-cell lymphoma 2; NF-κB, nuclear factors-κB; PSD-95, postsynaptic density protein-95; AR, adrenergic receptor; TRPV1, transient receptor potential vanilloid subtype 1; NMDAR, N-methyl-d-aspartate receptor; CaM, calmodulin; CaMKIV, calmodulin-dependent protein kinase type IV; CREB, cyclic adenosine monophosphate response elements binding protein; DA, dopamine; Ach, acetylcholine; 5-HT, 5-hydroxytryptamine; TLRs, Toll like receptors; PAMPs, pathogen associated molecule pattern; MyD88, bone marrow differentiation factor 88; Hspb1, heat-shock protein β1; pERK, phospho-extracellular signal-regulated kinase; CBF, cerebral blood flow; RNS, reactive nitrogen; ROS, reactive oxygen species; NO, nitric oxide; MDA, malondialdehyde; NOS, NO synthase; GSH-Px, glutathione peroxidase; TXNIP, thioredoxin-interacting protein; Trx, thioredoxin.

In conclusion, the effects and mechanisms of acupuncture on PSCI involves anti-neuronal apoptosis, promoting synaptic plasticity, alleviating central and peripheral inflammatory reactions, and regulating brain energy metabolism disorders (including improving cerebral blood flow, glucose utilization and mitochondrial structure and function, etc.), etc. The therapeutic effects and mechanisms of acupuncture on PSCI summarized in this review provides scientific and reliable evidence for acupuncture clinical application for PSCI (Figure 2).

## Author contributions

All authors contributed to data collection, analysis, drafting and revising the article, and gave final approval of the version to be published, agreed to the submitted journal.
